# To Be a Sportsman? Sport Participation Is Associated With Optimal Academic Achievement in a Nationally Representative Sample of High School Students

**DOI:** 10.3389/fpubh.2021.730497

**Published:** 2021-09-16

**Authors:** Sitong Chen, Xiaoyun Li, Jin Yan, Zhanbing Ren

**Affiliations:** ^1^Department of Physical Education, Shenzhen University, Shenzhen, China; ^2^Department of Physical Education, Shenzhen University, Shenzhen, China; ^3^Department of Physical Education Teaching, Zhongshan the Second Middle School, Zhongshan, China; ^4^Priority Research Centre in Physical Activity and Nutrition, School of Education, University of Newcastle, Newcastle, NSW, Australia

**Keywords:** youth, sports participation, academic outcome, survey, movement behavior

## Abstract

In the present study, the relationship between academic achievements and participation in a sports team in adolescents has been identified using nationally representative data. The study sample was created by referring to the U.S. Youth Risk Behavior Surveillance 2019 cycle, of which were eligible samples in the current study. A self-reported questionnaire was used to assess the participation in sports (0, one, two, three or more teams) and academic performance (mostly A, mostly B, mostly C, mostly E, mostly F). Controlling variables included sex, age, grade, race/ethnicity, adherence to physical activity, sleep guidelines and screen time, respectively. A binary regression model with an odds ratio (OR) at 95%CI confidence interval was performed to examine the association between sports team participation (0 teams as reference) and academic performance (combination of mostly C, E, F as reference). Results showed that compared with study participants with no participation in any sports teams, participating in one, two, three or more teams were more likely to self report better academic performance (1 teams: odds ratio [OR] = 1.48; two teams: OR = 2.34; three or more = 2.72), demonstrating a dose despondent association. This dose-dependent association varied slightly across sexes and grades. In conclusion, consistent with previous studies, the current study confirmed the positive roles of sport participation on academic outcomes in adolescents. Sex- and grade-specific strategies should be considered for academic-relevant promotion.

## Introduction

As a modality of physical activity, sports participation has been recognized as an important approach to promote healthy behaviors and overall development in children and adolescents (1–3). According to several research, children and adolescents can benefit from participation in sports (3–5). Specifically, the Iowa Bone Development Study indicated that sports participation could predict later sufficient physical activity (6). Several recently published research articles using longitudinal design have demonstrated a potentially negative association between greater sports participation and fewer symptoms of depression and anxiety in adolescents (1, 7, 8). Vella et al. (9) concluded that sports participation was positively correlated with the adherence to screen time and physical activity guidelines as well as fruit and vegetable consumption guidelines. Moreover, evidence has suggested that sports participation may be a feasible approach to reduce the odds of obtaining an unhealthy weight in children and adolescents (10, 11). These studies have stressed the prominent functions of sport participation in improving physical and psychological health and social development in children and adolescents.

Furthermore, in addition to the benefits of sport participation, recent studies have also begun focusing on the relationship between academic performance and sports participation (12, 13). The available research suggests a positive association exists between participation in sports and the academic performance of both children and adolescents. (14, 15). For example Burns et al. (16) used data of a nationally representative sample that exhibited a positive dose-dependent relationship between participation in team sports and academic performance (self-reported A's and B's). In addition Burns et al. (17) indicated that adolescents with sports participation were likely to report better academic performances. The cross-sectional evidence confirmed the positive roles of sports participation on academic performance. A 2-year longitudinal study also supported the evidence from cross-sectional studies (18). In this study, the authors suggest that sports participation provides significant benefits for improving academic achievement (18).

Across literature, the positive association between participation in sports and academic performance in children and adolescents has been confirmed. However, some research gaps remain that need to be addressed for upgrading the evidence in this research field. One of the greatest gaps is that there are a number of studies that failed to use nantionally representative sample (12, 19–21), which limits the generabsiabiliy of research findings. Another gap is that previous relevant studies have failed to explore whether the relationship between sports participation and academic performance varies due to demographics, such as sex or age. Answering this question is conducive to gaining deeper insights into the relationship between academic performance and sports participation, which in turn can lead to improved strategies to promote optimal academic performance. Consequently, this current study shall explore the association between academic performance and sports participation using the nationally representative sample based in the U.S. Youth Risk Behavior Surveillance data of the 2019 cycle. Additionally, the relationship between sports participation and academic performance when considering sex and age shall be explored.

## Methods

### Study Design and Participants

The YRBS project was a national survey that was established in 1990 (at a two-year interval) to understand the health-related behaviors in youth at grades 9–12 in the United States. Data for this present cross-sectional survey collected in 2019 was analyzed in 2021. A sampling design based on the multistage cluster was utilized to generate nationally representative results. All students from these classes met the standard for participation in the survey. More details concerning YRBS procedures can be found by accessing the Centers for Disease Control and Prevention website. Explicitly, the complete data incorporates the eligible participants, a total of 6,946 participants aged 14-17 years, for all relevant variables included in this study.

### Measures

Individual Demographics-A self-report questionnaire was used to collect data on study participants, which includes personal information such as sex (female or male), grades (9, 10, 11 and 12), and ethnicity/race (Black/African American, White, Latino/Hispanic or other), as well as body height and weight for determination of body mass index (BMI).

Sports participation-One question asked study participants to report the number of their team sports participation; “During the last year, how many team sports did you engage in?”. The possible answers included 0, 1, 2 and 3 or more teams.

Behavioral covariates-Based on the previous studies (17, 18, 22–27), some selected behavioral factors were covariates of this study. Study participants answered an item that asked: “Over the last week, how many days did you spend on sports exercises for at least 60 minutes per day?”. Response options included “0–7 days”. Responses were dichotomized to determine whether participants met the physical activity guidelines that recommend at least 1 hour of moderate-to-strenuous physical activity (23). Participants responded to two items that asked: “On average, at day time, how many hours do you spend on the video and computer games, watch TV or use the computer for things in addition to the schoolwork?”. Responses were dichotomized to represent whether participants met the guideline, that is, ≤ 2 hours spent on screen time per day (23). In addition, study participants responded to one item that asked: “On average, at night-time, how long do you spend on sleep?”. Responses were dichotomized to represent whether participants met the guideline, i.e., sleep for 8–10 hours every night among adolescents aged 14–17 (23).

Academic achievement-The students have been told to provide the average scores they achieve in school. The response selections include mostly F's, D's, C's, B's and mostly A's. The response of mostly A's was treated as first-class AA, while others were treated as non-first-class AA. This item was used frequently in previous literature and surveys (16, 28), despite no convincing evidence of its reliability and validity.

### Statistical Analysis

All analyses in this research was conducted using Stata 16.1 (Stata Corporation, College Station, TX, USA). Missing data have been addressed by complete cases before formal analysis. Commands for the survey study (“prefix: survey” in STATA) based on the complex survey design provided by YRBS were used to generate nationally representative estimates of results in this study. Weights of the population were included to regulate the unequal possibility of selection. Descriptive statistics were conducted to report the characteristics of the study participants. A bio-nominal regression model was performed to examine the association between team sports participation (0 teams as the reference group) and AA (non-first-class as the reference group). Three models were established. The first model was the crude model that only examined the association between team sports participation and first-class AA without any adjustments. The second regression model adjusted the variables of sex, age, grade, race/ethnicity and BMI. The third model included all the relevant variables to perform a final evaluation of the relationship between participation in team sports and AA. Regression results by sex or grade-specific were also presented. For all the analyses, the alpha level is *p* < 0.05 for statistical significance.

## Results

Table 1 displays weighted and unweighted results for the characteristics of the study sample. In detail, this study sample consisted of 3,662 females and 3,284 males. The percentage of 17 year-olds, 16 year-olds, 15 year-olds and 14 year-olds sample was 12.6, 29.0, 31.2 and 27.2%, respectively. More than half of the study sample was of white race (% weighted = 52.6). The average value of BMI was 23.5 (standard deviation = 5.3). Of the study samples, 23.6% of them met the physical activity guidelines, 31.8% of them met the screen time guidelines and 21.6% of them met the sleep guidelines. The percentage of the study sample engaging in one team, two teams and three or more teams for sports participation was 27.4, 18.6 and 13.5%, respectively. Nearly 80% of the study sample reported grades of A's or B's. Further information on weighted results can be found in Table 1.

**Table 1:** Descriptive results of the study sample, both weighted and un-weighted.

		**Unweighted count**	**Unweighted percentage**	**Weighted percentage**
Sex				
	Females	3662	52.7	50.5
	Males	3284	47.3	49.5
Age				
	14 years old	877	12.6	13.1
	15 years old	2014	29.0	28.9
	16 years old	2168	31.2	29.9
	17 years old	1887	27.2	28.2
Grade				
	9^th^	2080	29.9	30.3
	10^th^	2193	31.6	29.4
	11^th^	1842	26.5	27.4
	12^th^	831	12	12.9
Race/Ethnicity				
	White	3520	50.7	52.6
	Black or African American	910	13.1	9.4
	Hispanic/Latino	1735	25.0	27.5
	All other races	781	11.2	10.4
Body mass index (mean ± sd)						23.5 ± 5.3	/	23.6 ± 5.1
Physical activity guidelines				
	Not meeting	5306	76.4	74.8
	Meeting	1640	23.6	25.2
Screen time guidelines				
	Not meeting	4735	68.2	68.6
	Meeting	2211	31.8	31.4
Sleep guidelines				
	Not meeting	5447	78.4	78.1
	Meeting	1499	21.6	21.9
Sports team participation				
	0 teams	2812	40.5	39.1
	1 team	1904	27.4	27.0
	2 teams	1292	18.6	19.1
	3 or more teams	938	13.5	14.7
Academic performance (grades)				
	Not A's or B's	1404	20.2	20.2
	A's or B's	5542	79.8	79.8

*Sd, standard deviation; Physical activity guidelines, at least 1 hour for moderato to strenuous physical activity every day is recommended; Guidelines for screen timer, no more than 2 hours spent on screen every day is recommended; Sleep guidelines, 8–10 hours for sleep duration per day is recommended*.

Table 2 shows the results obtained through multiple logistic regression, which reveals the relationship between independents and academic performance (A's or B's). Three models were established. Specifically, in model 1, only team sports participation was included in the analysis and the results showed that greater engagement in team sports participation was more likely to engender better academic performance. Compared with engaging in no team sports participation, engaging in one team, two teams and three or more teams was 1.52 (95% CI: 1.29–1.79), 2.31 (95% CI: 1.87–2.85) and 2.77 (95% CI: 2.05–3.75) times greater to report A's or B's grades in academic performance. In model 2, when controlling for sex, age, grade, race/ethnicity and BMI, the association between sports team participation and academic performance remained significant. When adjusting the variables such as sleep, screen time and physical activity, the relationship between participation on a sports team and academic performance was still significant (OR for 1 team = 1.48, 95% CI: 1.25–1.76; OR for 2 teams = 2.34, 95% CI: 1.84–2.97; OR for 3 teams = 2.72, 95% CI: 1.96–3.79). In all the three regression models, a dose-dependent relationship between participation on a sports team and academic performance was found, indicating an increased likelihood of engendering better academic performance with more sports team participation.

**Table 2:** Parameter estimates obtained from the multiple logistic regression model weighted based on the outcome of academic performance (A's and B's).

		**Model 1**			***p***	**Model 2**			***p***	**Model 3**			***p***
		**OR**	**95%CI**			**OR**	**95%CI**			**OR**	**95%CI**		
Sports team participation													
	0 teams	Ref				Ref				Ref			
	1 team	**1.52**	**1.29**	**1.79**	0.00	**1.49**	**1.27**	**1.75**	0.00	**1.48**	**1.25**	**1.76**	0.00
	2 teams	**2.31**	**1.87**	**2.85**	0.00	**2.36**	**1.87**	**2.96**	0.00	**2.34**	**1.84**	**2.97**	0.00
	3 or more teams	**2.77**	**2.05**	**3.75**	0.00	**2.77**	**1.99**	**3.86**	0.00	**2.72**	**1.96**	**3.79**	0.00
Sex													
	Females					**1.89**	**1.60**	**2.23**	0.00	**1.91**	**1.63**	**2.24**	0.00
	Males					Ref				Ref			
Age													
	14 years old					Ref				Ref			
	15 years old					0.90	0.65	1.23	0.32	0.91	0.65	1.27	0.21
	16 years old					0.82	0.52	1.28	0.12	0.82	0.52	1.30	0.14
	17 years old					0.69	0.39	1.22	0.15	0.69	0.39	1.24	0.23
Grade													
	9th					Ref				Ref			
	10th					1.01	0.72	1.42	0.35	1.02	0.73	1.43	0.08
	11th					1.15	0.75	1.76	0.47	1.20	0.78	1.83	0.14
	12th					1.68	0.89	3.17	0.33	1.76	0.94	3.31	0.56
Race/Ethnicity													
	White					Ref				Ref			
	Black or African American					0.57	0.42	0.77	0.30	0.58	0.43	0.77	0.35
	Hispanic/Latino					0.64	0.51	0.80	0.37	0.64	0.51	0.80	0.27
	All other races					**1.46**	**1.16**	**1.83**	0.00	**1.47**	**1.17**	**1.85**	0.00
Body mass index (mean ± sd)																			**0.97**	**0.95**	**0.98**	0.00	**0.97**	**0.95**	**0.98**	0.00
Physical activity guidelines													
	Not meeting									Ref			
	Meeting									0.97	0.77	1.21	0.99
Screen time guidelines													
	Not meeting									Ref			
	Meeting									1.14	0.96	1.35	0.14
Sleep guidelines													
	Not meeting									Ref			
	Meeting									**1.54**	**1.30**	**1.82**	0.00

*Sd, standard deviation; Physical activity guidelines, at least 1 hour for moderato to vigorous physical activity per day is recommended; Screen time guidelines, no more than 2 hours of screen time per day is recommended; Sleep guidelines, 8–10 hours for sleep duration per day is recommended. Bold values denote statistically significant*.

Table 3 demonstrates the results for the relationship between sports team participation and academic performance by sex and grade after adjusting for all the other controlling variables. Specifically, in Table 3, compared with male adolescents not involving in any sport participation teams, those who joined in more teams showed greater likeliness of reporting better academic achievement, and this significant association was dose-dependent. According to Table 3, the significant association between participation in sports team and better academic performance was not dose dependent. In females, those who participated in two team sports had the highest odds (OR = 2.97, 95%CI: 1.98–4.46) of reporting better academic performance compared with involvement in zero team sports. Table 3 illustrates the results of the relationship between sports team participation and better academic performance in the study sample with different grades. In grade 9 students, only having two or three team sports (2 teams OR = 2.13, 95%CI: 1.42–3.20; 3 or more teams OR = 3.42, 95%CI: 1.91–6.12) were more likely to report better academic performance. In grade 10 and 11 students, participating in team sports was associated with better self-reported academic performance. However, in grade 12 students, only participating in three or more team sports was associated with better academic performance (OR = 4.44, 95%CI: 1.38–14.28).

**Table 3:** Relationship between academic performance and participation in sports team by sex and grade.

		**1 team**			***p***	**2 team**			***p***	**3 or more teams**			***p***
		**OR**	**95% CI**			**OR**	**95% CI**			**OR**	**95% CI**		
Sex													
	Females	1.65	1.22	2.23	0.00	2.97	1.98	4.46	0.00	2.63	1.47	4.71	0.00
	Males	1.37	1.05	1.78	0.02	2.05	1.52	2.76	0.00	2.73	2.01	3.69	0.00
Grade													
	9th	1.20	0.89	1.61	0.20	2.13	1.42	3.20	0.02	3.42	1.91	6.12	0.00
	10th	1.56	1.20	2.01	0.04	2.60	1.93	3.51	0.00	1.98	1.11	3.54	0.00
	11th	1.78	1.25	2.53	0.01	2.35	1.34	4.12	0.00	2.62	1.60	4.30	0.00
	12th	1.44	0.93	2.23	0.052	2.08	0.96	4.54	0.33	4.44	1.38	14.28	0.02

*Reference group, 0 teams; models were controlled for variables age, grade, race/ethnicity, sleep, screen time, physical activity, body mass index*.

## Discussion

The knowledge to date related to a few similar studies shows that the current research aimed to examine the association between team sports participation and academic performance, adding accumulating evidence to the literature and confirming the positive roles of sports team participation on cognitive outcomes in adolescents. Moreover, the current study explored sex and grade differences in the relationship between participation on team sports and academic performance. In sum, in the present study, it is found that participation on team sports was positively correlated with academic performance in adolescents, and this association remained significant regardless of sex and grade.

The current research findings that participation on team sports was independently correlated with academic performance is supported by previous studies (16, 23, 24, 29, 30). For example Fox et al. (23) found that team sports participation was positively correlated with higher grade point averages in high school students, which is comparable to this study sample. A recently published study by Burns et al. (16) also suggested that more team sports participation in adolescents was associated with better academic performance. In addition, some longitudinal studies also support the research findings (18). Possible explanations for the positive relationship between participation in sports team and better academic performance in adolescents include: (1) participation in team sports promotes a student's sense of identity with school and the associated school culture or recognized values, which could, in turn, improve the students “academic performance (29): (2) team sports participation can enhance adolescents” psychological resilience, self-esteem, self-discipline and overall life skills (1); these skills have been recognized as incentives to better academic performance. However, the current study failed with academic performance to further explore the underlying mechanism linking sports team participation in adolescents, which should be addressed in future studies. Data shows that nearly 60% of U.S. adolescents have participated in team sports activities, which is a meaningful result. Based on this study finding, many U.S. adolescents are likely to have opportunities to enhance academic performance. However, maintaining sport participation by adolescents is a challenging matter and this could be a barrier for adolescents to gain more physical, mental and social benefits (31, 32).

Results of the current study showed that the association between participation in sports team and academic performance was dose-dependent, in that involvement in more than one team sports had a more prominent relationship with academic performance compared with participation in fewer team sports (see Table 2). This research finding can be used for encouraging adolescents to engage in multiple team sports. To date however, there are no well-accepted interpretations concerning the dose-dependent relationship between participation on team sports and academic performance. This offers a direction for future research and has some meaningful implications. Owing to the varied operationalized definitions of team sports participation, it is possible that team sports participation in this study could be either community-based or school-based. Thus, designing interventions that increase participation in community-based and school-based sports activities may engender further opportunities to secure more benefits. In the long run, it is a feasible approach that promoting sport participation for adolescents can benefit their overall development and health.

It was discovered that sex and grade differences somewhat impacted the association between participation in team sports and academic performance, which was not observed in the overall sample. Specifically, the current study found that the association between participation in team sports and academic performance couldn't be described as dose-dependent in females and 10^th^ graders. Moreover, unlike the previous research findings based on the overall sample, engaging in one sports team, in 9^th^ graders and one or two team sports in 10^th^ graders, was not significantly associated with better self-reported academic performance. These variations are not further clarified in this study because there was no further supporting evidence. However, from a practical perspective, these findings may be conducive to guiding interventional strategies for adolescents with different characteristics (e.g., females and higher graders).

It has been documented that many factors influence academic performance in adolescents. This study found that females and adolescents of all races or ethnicities were more likely to report better academic performance. However, these are unmodifiable factors in the real world. Moreover, this study found that BMI and more sleep was also correlated with better academic performance in adolescents. Specifically, higher values of BMI were negatively associated with better academic performance, but appropriate sleep duration was positively correlated with better academic performance. Previous studies have demonstrated that appropriate sleep duration may be a promoting factor for better academic performance in adolescents (24, 33), as optimal good quality sleep duration can improve cognitive functions, which in turn enhance the academic performance of adolescents. In contrast, higher values of BMI may negatively influence academic performance in adolescents. This research finding is consistent with prior research (34). Considering the adverse health effects of higher BMI values, conducting healthy body weight management is required.

### Practical Implications and Research Direction

This research provides additional confirmatory evidence of the positive role of team sports participation on academic performance in adolescents, which also supports prior research findings (16, 18, 23). When considering this study findings and the merits of sports team participation, school-based interventions incorporating sports team participation can be applied to promote better academic performance in adolescents. Notably, sex- or age-specific interventions are recommended when designing strategies to promote academic performance. However, research thus far on sport participation remains scant, which should be explored in the future for a deeper understanding of sport participation in adolescents, such as correlates of sport participation. As sports participation is a modality of physical activity, the research framework of physical activity epidemiology can be used for research into sports participation. Compared with research on overall physical activity, research on sport participation needs more attention.

### Study Limitations and Strengths

Some study limitations must be mentioned for a better understanding of the research findings. Firstly, there are limitations with self-reporting of academic performance because it is prone to recall bias and social desirability. Additionally, due to the design of the cross-sectional study, a causal association between academic achievements and participation on team sports could not be drawn. However, this study does have some strengths worth mentioning. A key strength of the current study was to collect data about both sports team participation and physical activity. In doing so, the independent contribution of team sports participation to academic performance can be examined. Another strength of this study has been the study sample which was nationally representative. This could enlarge the generalization of the research findings.

## Conclusion

This study has offered evidence on the positive association between academic performance and participation in team sports using a nationally representative sample in the US. This study also added evidence on the sex and grade variations in the relationship between academic performance and participation in a sports team. Future studies should negate or confirm these research findings using an improved study design that can provide more reliable clinical implications.

## Data Availability Statement

The raw data supporting the conclusions of this article will be made available by the authors, without undue reservation.

## Author Contributions

SC, XL, and JY: study design and manuscript draft. SC and ZR: data analysis and manuscript revision. All authors contributed to the article and approved the submitted version.

## Funding

ZR was funded by Research Foundation for Young Teacher of Shenzhen University (grant number QNJS0274); High-level Scientific Research Foundation for the Introduction of Talent of Shenzhen University (grant number RC00228). SC was funded by the National Social Science Foundation of China (grant number 19CTY010).

## Conflict of Interest

The authors declare that the research was conducted in the absence of any commercial or financial relationships that could be construed as a potential conflict of interest.

## Publisher's Note

All claims expressed in this article are solely those of the authors and do not necessarily represent those of their affiliated organizations, or those of the publisher, the editors and the reviewers. Any product that may be evaluated in this article, or claim that may be made by its manufacturer, is not guaranteed or endorsed by the publisher.
